# Genomic Characterization of *Orf Virus* Strain D1701-V (*Parapoxvirus*) and Development of Novel Sites for Multiple Transgene Expression

**DOI:** 10.3390/v11020127

**Published:** 2019-01-30

**Authors:** Hanns-Joachim Rziha, Mathias Büttner, Melanie Müller, Ferdinand Salomon, Alena Reguzova, Dominic Laible, Ralf Amann

**Affiliations:** 1 Interfaculty Institute for Cell Biology, Department of Immunology, Auf der Morgenstelle 15, 72076 Tübingen, Germany; achim.rziha@ifiz.uni-tuebingen.de (H.-J.R.); melanie.mueller@uni-tuebingen.de (M.M.); ferdinand.salomon@uni-tuebingen.de (F.S.); alena.reguzova@uni-tuebingen.de (A.R.); do.laible@googlemail.com (D.L.); 2 Institute of Immunology/Molecular Pathogenesis, Center for Biotechnology and Biomedicine, College of Veterinary Medicine, Leipzig University, 04103 Leipzig, Germany; mathias.buettner@uni-leipzig.de

**Keywords:** viral vector, *parapoxvirus*, *Orf virus*, ORFV, gene deletion, attenuation, recombinant ORFV, Vero cell adaptation

## Abstract

The *Orf virus* (ORFV; *Parapoxvirus*) strain D1701 with an attenuated phenotype and excellent immunogenic capacity is successfully used for the generation of recombinant vaccines against different viral infections. Adaption for growth in Vero cells was accompanied by additional major genomic changes resulting in ORFV strain variant D1701-V. In this study, restriction enzyme mapping, blot hybridization and DNA sequencing of the deleted region s (A, AT and D) in comparison to the predecessor strain D1701-B revealed the loss of 7 open reading frames (ORF008, ORF101, ORF102, ORF114, ORF115, ORF116, ORF117). The suitability of deletion site D for expression of foreign genes is demonstrated using novel synthetic early promoter eP1 and eP2. Comparison of promoter strength showed that the original *vegf-e* promoter Pv as well as promoter eP2 display an up to 11-fold stronger expression than promoter eP1, irrespective of the insertion site. Successful integration and expression of the fluorescent marker genes is demonstrated by gene- and insertion-site specific PCR assays, fluorescence microscopy and flow cytometry. For the first time ORFV recombinants are generated simultaneously expressing transgenes in two different insertion loci. That allows production of polyvalent vaccines containing several antigens against one or different pathogens in a single vectored ORFV vaccine.

## 1. Introduction

Viral vector vaccines represent excellent inducers of cell-mediated and humoral immune responses. Therefore, intensive investigations aim to improve the use of several virus families as safe and efficient viral vectors, not only against diverse infectious diseases but also against tumours or for gene therapy [1,2,3,4]. In particular recombinant poxviruses represent popular live vectors owing to (i) their stability, (ii) their large genomic size allowing flexible integration of multiple foreign genes, (iii) their exclusive cytoplasmic gene expression independent from the host cell machinery and therefore, constitute no risk of gene integration into the host genome leading to insertional cellular gene inactivation, (iv) the very low mutation rates of the recombinants’ genome and (v) most importantly their ability to stimulate long-lasting transgene-specific B- and T-cell immunity (for review [5,6,7]). Less attenuated, replication competent *Vaccinia virus* (VACV) vectors provoked some inadvertent complications after immunization [4,8] and thus, highly attenuated poxvirus strains were developed from *Canarypox*, *Fowlpox* or VACV, which are replication-deficient in mammalian cells [2,3,8,9]. The *modified Vaccinia Ankara* (MVA) was established as a remarkably attractive and successful vector virus system resulting in various protective vaccines for use in veterinary and human medicine [10,11]. However, concerns exist that those highly attenuated, replication deficient vectors induce an immune response, which might be less effective and less lasting compared to their replication competent counterparts. Therefore, optimized poxvirus vectors are desirable that induce potent, protective and long-lasting immunity [5,12,13].

Lately we reported on a novel, promising virus vector system for the expression of different foreign antigens using the *Orf virus* (ORFV), the type species of the genus *Parapoxvirus* of the poxvirus subfamily *Chordopoxvirinae*. Infection with the dermotropic ORFV occurs via the broken skin causing localized contagious pustular dermatitis in sheep and goats (for reviews see [14,15]). One remarkable property of ORFV is the very restricted host range both in vivo and in vitro [16], which can also complicate the virus propagation. Another distinctive property of ORFV is its lack of systemic spread even after intravenous injection or in immunosuppressed animals [14,17,18]. Moreover, the lack of ORFV neutralizing antibodies in infected animals is an uncommon feature [19,20], however, advantageous for repeated vector application. Our virus vector system is based upon the ORFV strain D1701 possessing strongly reduced pathogenicity. Similar to VACV vector MVA the ORFV strain D1701 was attenuated by multiple in vitro passages in primary ovine and bovine cell cultures that finally led to a registered live vaccine against Orf disease [21]. Previously ORFV D1701 has been shown to possess exceptionally strong and fast stimulation of innate cellular immune mechanisms effective against a variety of pathogens in several species [22,23,24]. Used in an inactivated form it is applied as a non-specific preventive and metaphylactic immune modulator in veterinary medicine [25,26,27]. The result of its attenuation by cell culture passaging was a remarkable genomic rearrangement ensuing deletion, duplication and transposition of genes located in the genomic termini [28]. Thereafter, the virus, obtained from a bovine kidney cell line BK-KL3A [29] designated D1701-B, was further adapted for growth in the African green monkey cell line Vero resulting in the D1701-V virus. Even after immunosuppression D1701-V is completely apathogenic in the natural host sheep and the further reduction in virulence is presumably associated with additional genomic deletions [14,18,30,31].

All these properties favour ORFV D1701-V as a promising live virus vector platform for delivering various heterologous microbial antigens to the immune system. The possibility to insert foreign genes into the *vegf-e* (V) locus, which encodes an important virulence factor [32,33,34], allowed us for the first time the generation of ORFV recombinant vaccines that mediate excellent and long-term protective immune responses against diverse viral infections in different hosts without the need of an adjuvant such as demonstrated in mouse, dog, cat, cattle, swine or rabbit [35,36,37,38,39,40,41,42]. *Parapoxvirus* replication is restricted to the cytoplasm and the temporarily regulated gene expression is divided into immediate early, early, intermediate and late phases as characteristic for poxviruses [7,43,44,45,46]. In all our ORFV recombinants until now we utilized the authentic early promoter of the substituted *vegf-e* gene (Pv) enabling strong early transgene expression without the need of ORFV genome replication or production of infectious virus and therefore, exhibiting properties of a replication-deficient vaccine. During these studies we found that expression of several foreign genes successively inserted into the *vegf-e* (V) locus and controlled only by the Pv promoter was not as strong as after regulation of each transgene by a distinct promoter [47]. Improvements on the utility of the ORFV vector system are desirable in terms of providing additional insertion sites for more foreign genes associated with new early ORFV promoters. Also an acceleration of the selection procedure of recombinant ORFV would be advantageous, because the integration of foreign genes relies on intermolecular homologous recombination with transfer plasmids transfected into virus infected cells [48], which requires tedious selection by multiple rounds of picking single virus plaques. The use of fluorescent marker genes was reported to facilitate the selection process for the isolation of virus recombinants [49,50], for example, by red-to-green gene swapping [51], which was also the basis for a flow cytometric selection and purification protocol of VACV MVA recombinants [52].

The present work describes the exact delimitation, fine mapping and DNA sequencing of the three regions deleted in the genome of D1701-V, which were charted roughly earlier [18] and are now designated A, AT and D, respectively. Comparative genomic analyses between D1701-V and its precursor D1701-B revealed which genes or parts thereof have been lost during adaption for growth in Vero cells. The construction of novel transfer plasmids is described to enable stable early expression of several foreign genes in the new insertion locus D. Fluorescent marker gene based strategy is used for the generation of ORFV recombinants allowing multigene expression not only in the D but also in the V locus of the ORFV genome. To this end new synthetic ORFV early promoters were designed and their expression strength compared. Conclusively, the presented data demonstrate now an important improvement of our ORFV vector platform for the successful generation of multivalent vaccines.

## 2. Materials and Methods

### 2.1. Cells, Virus

D1701-B originated from the ORFV field isolate D1701 after multiple passages in foetal lamb kidney or lung cells before adapted to grow in cell line BK-KL3A [29]. The virus D1701-BK50 was additionally passaged 50-times in BK-KL3A cells using a multiplicity of infection (moi) of approx. 0,1. The Virus D1701-V was three times plaque-purified after 45 passages of D1701-B in the monkey kidney Vero cell line. Virus propagation, titration and cell cultivation were performed in Vero cells or in foetal bovine oesophageal cells (KOP, RIE 244, cell culture collection of the Friedrich-Loeffler-Institute, Federal Res. Inst. Animal Health, Island of Riems, Germany) as described [28,31,53]. ORFV gene expression was arrested in the early phase by adding 40 µg Cytosine arabinoside (AraC) per mL medium 30 min before and during infection.

### 2.2. DNA Preparation, Restriction Digests, Southern Blotting

ORFV DNA was isolated, restriction enzyme digested and blot hybridized as described earlier [28,31]. For ORFV recombinant screening, DNA was prepared from infected cells showing approximately 80% cytopathogenic effect (cpe) according to the manufacturer’s protocol (Master Pure DNA isolation kit from Illumina Cam. Lim.). Quick plasmid DNA preparation was achieved as reported [31] or using Genelute Plasmid Miniprep kit (Sigma-Aldrich, Taufkirchen, Germany). For nucleofection the plasmids were purified with Midi DNA preparation kit (Qiagen, Hilden, Germany).

### 2.3. DNA Sequencing

Nucleotide sequence of the various cloned fragments of D1701-B and D1701-V DNA was obtained by primer walking and accelerated sequencing of DNA fragments by the use of transposon-generated template system as described earlier [28,54]. The new transfer plasmids were Sanger sequenced by Eurofins GATC Biotech (Konstanz, Germany).

### 2.4. Novel Synthetic ORFV Early Promoter eP1 and eP2

Based on the reported consensus sequences defining a 15–16 bases comprising critical core region of poxviral early promoter [7,46,55,56,57] we designed the synthetic early promoter eP1 and eP2, respectively. Directly adjacent to the critical promoter core motif, which is given in Table 1, a multiple cloning site (MCS) of 30–50 nucleotides (nt) is additionally synthesized to cover varying restriction cleavage sites for insertion of foreign genes. Finally, an 80 nt long spacer region (SP) separating promoter eP1 and eP2 is integrated to ensure strong foreign gene expression from each promoter. The SP sequence begins with 3 consecutive T5NT motifs for termination of early gene transcription (Table 1) to prevent overlapping transcription and thus, reduced expression of an upstream inserted transgene [58].

Moreover, two synthetic tandemly repeated early promoters were constructed. Downstream of Pv in plasmid pV-mCherry five joined core elements of Pv (5’-CAAAATGTAAATTATA-3’), separated by CCGGT from each other, were inserted, which resulted in the recombinant virus D1701-V-5xPv-Cherry (abbreviated V-5xPv-Cherry). Another construct was prepared by adding five eP2 core elements (5′-AAAAATTGAAATTCTA-3′), separated by GGCCT from each other, downstream of Pv in pV-mCherry to obtain the virus D1701-V-5xeP2-Cherry (V-5x2-Cherry). These ORFV recombinants were intended to evaluate the influence of tandemly repeated early promoter elements on the strength of transgene expression in the V locus of ORFV D1701-V.

### 2.5. Construction of Transfer Plasmids

For integration of transgenes into the D locus transfer plasmid pD12-mCherry was generated as illustrated in Figure S1. First, plasmid pV-mCherry (Figure S1A) was digested with the restriction endonuclease *Sma*I, re-ligated and subsequently digested with *Sal*I. Upon re-ligation plasmid pV-mCherry-Sma+Sal was obtained (Figure S1B). The left homology arm Del2-L (919 bp in size) containing singular *Sma*I and *Age*I restriction sites was synthesized (Mr.Gene). It covers 653 bp of the D1701-B genome followed by multiple cloning sites (MCS), the early promoter eP1 and eP2 separated by the spacer region (SP; nucleotide sequences see also Table 1). The right homology arm Del2-R (731 bp) was also synthesized (Mr. Gene) containing singular *Not*I and *Sal*I restriction sites and 655 nt of the D1701-B genome (Figure S1B). To guarantee strong early expression several poxviral early stop motifs T5NT were placed after the MCS for proper foreign gene termination. As depicted in Figure S1B the left arm Del2-L and the right arm Del2-R were ligated into pV-mCherry-Sma+Sal to embrace the *mCherry* gene. The resulting plasmid pD12-mCherry (Figure S1C) can be used to generate D1701-V recombinants expressing the *mCherry* gene in locus D under control of the early promoter eP2. Moreover, this plasmid serves for further plasmid constructions by inserting foreign genes directly downstream of eP1 as exemplarily outlined in the Results part.

Plasmid pV-AcGFP was created by cloning the *Eco*RI-*Bam*HI fragment of pAcGFP1 (TaKaRa Clontech, USA) into the ORFV transfer plasmid pdV-Rec as described earlier [31,37]. These plasmids could also be used for the insertion of each marker gene into the *vegf-e* locus (V insertion site) of D1701-V [31].

### 2.6. Nucleofection

Transfer plasmid DNA was injected into D1701-V infected cells by nucleofection as detailed recently [31] using the nucleofector device (Lonza, Köln, Germany) and CLB transfection system (Biozym, Hamburg, Germany).

### 2.7. Western Blotting

Specific protein detection from cell lysates was achieved by Western blotting and enhanced chemiluminescence as reported [40]. The monoclonal antibody 4D9 recognizes the late major envelope protein F1L (ORF059) of ORFV [59], the antibody specific for cellular ß-actin was purchased from Sigma-Aldrich.

### 2.8. Flow Cytometry

To detect fluorescent marker gene expression after infection Vero cells were trypsinized, washed twice with PBS (phosphate buffered saline) and analysed by flow cytometry. Non-infected cells were included as negative control. Viability staining of infected cells was performed with Zombie Aqua™ dye (BioLegend, San Diego, USA) for 30 min at room temperature. Each sample (0.5 × 10^5^ cells) was measured with BD LSRFortessa (BD Biosciences, Heidelberg, Germany) and analysed using the FlowJo software (FlowJo, LLC, Ashland, AL, USA). Data were gated on forward-scatter (FSC-A versus SSC-A), single side scatter (SSC-A versus SSC-H) and live (Zombie Aqua negative) cells.

In order to obtain comparable infection rates of Vero cells, flow cytometry was performed using dilutions (duplicates or triplicates) of Vero cells infected with the *AcGFP*- or *mChery*-expressing recombinants 20–24 h after infection. The infection rate represented by the percentage of fluorescent, infected cells was determined.

### 2.9. Polymerase Chain Reaction (PCR)

Identification and characterization of new ORFV recombinants was assisted by insert gene- and locus-specific PCR using 2× DreamTaq Green, 2× AmpliTaq Gold polymerase (Fisher Scientific, Schwerte, Germany) or Fast Gene Optima Taq polymerase (Nippon, Dueren, Germany) as described [31]. The used primers are listed in Table 1, the sizes of the amplicons are given in the text. PCR products are separated in horizontal Midori green (Nippon) stained agarose gel.

### 2.10. Selection of ORFV Recombinants

ORFV recombinants containing fluorescent marker genes were first pre-selected by flow cytometry [52] followed by limiting dilution cloning in 348-well plates (Perkin-Elmer, Rodgau, Germany) and final virus plaque purification to obtain homogeneous ORFV recombinants [31]. Identification of recombinant ORFV was additionally achieved by marker gene-specific PCR. The fluorescent transgene can be replaced by any foreign gene of interest leading to non-fluorescent, marker gene-free ORFV recombinants. Again, the selection of these new recombinants can be achieved by limiting dilution assays combined with foreign gene-specific PCR as described above.

## 3. Results

### 3.1. Detection and Mapping of Three Major Deletions in the D1701-V Genome.

**Figure 1:**
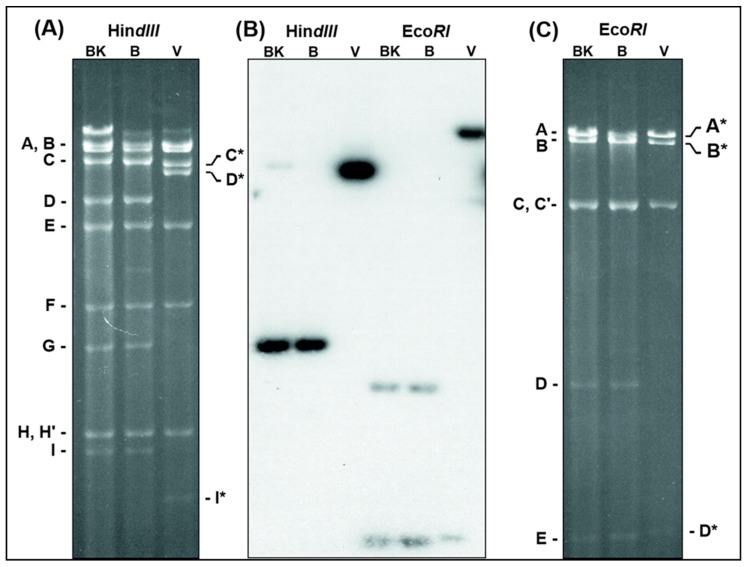
Changes of D1701-V DNA restriction fragment patterns. DNA of D1701-50BK (lanes BK), D1701-B (lanes B) and D1701-V (lanes V) was digested with Hin*dIII* or Eco*RI* and separated in a horizontal 0.8% agarose gel for 36 h. The digested DNAs were run in one identical gel, however, for clearer representation of the individual DNA fragments the photograph of the ethidium-bromide stained gel was split into the shown parts (**A**) and (**C**), respectively. (**B**) Demonstrates the X-ray after Southern blot hybridization of the gel with the ^32^P-labelled Hin*dIII*-fragment G of D1701-B. Those DNA fragments changed after Vero cell culture passaging are marked by a star.

**Figure 2:**
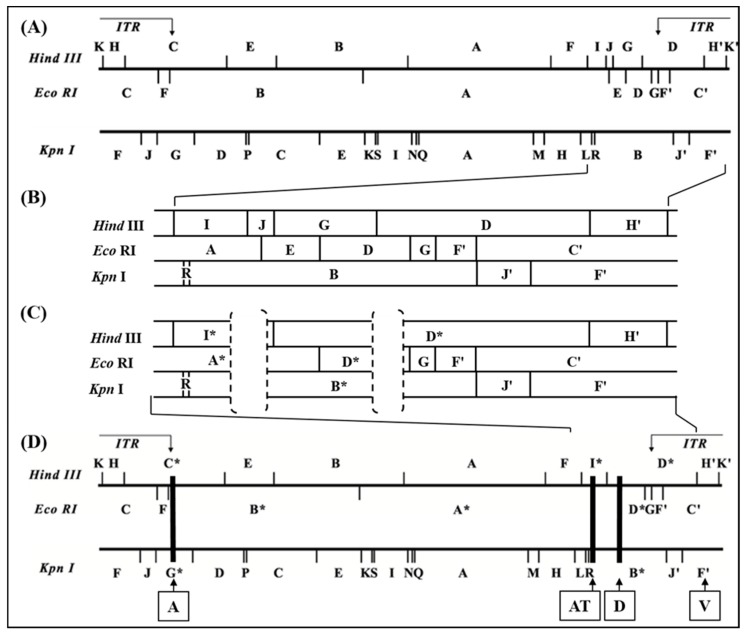
Map locations of the deleted parts of the D1701-V genome. The genomic map of the Hin*dIII*-, Eco*RI*- and Kpn*I*-fragments of D1701-B (**A,B**) and D1701-V (**C,D**) is depicted. ITR indicate the inverted terminal repeats at the genomic termini. In (**B**) and (**C**) the sequenced right-hand terminus of D1701-B and D1701-V is shown enlarged. The regions AT and D deleted in D1701-V are indicated by dashed brackets. Those DNA fragments affected by the genomic deletions are marked by stars. (**D**) The black rectangles indicate the deleted regions A, AT and D in D1701-V; in addition, the earlier described insertion site V (*vegf-e* gene) is indicated.

The original ORFV D1701-B represents a derivative of the plaque-purified attenuated ORFV vaccine strain D1701 as described by Mayr [21] and was used for further serial culture passages in Vero cells. After several blind passages ORFV characteristic cytopathogenic effects (cpe) appeared and the virus harvests stably reached titres exceeding 10^7^ plaque-forming units (pfu) per mL. Finally, the virus obtained from passage 45 was three times plaque-purified and designated D1701-V. No differences in size and morphology of the virus plaques could be observed between D1701-B and D1701-V when compared in KOP cells. In parallel, D1701-B was additionally passaged 50-times in BK-KL-3A cells (D1701-50BK). Viral DNA was isolated from D1701-V, D1701-50BK and D1701-B for comparative restriction enzyme analyses. Figure 1 shows the results after Hin*dIII* (Figure 1A) or Eco*RI* (Figure 1C) digest and Southern blot hybridization with the D1701-B Hin*dIII* fragment G as a ^32^P-labelled probe (Figure 1B). This demonstrates that the original Hin*dIII* fragment G of D1701-50BK and D1701-B with a size of 7.3 kbp was missing in the DNA of D1701-V but hybridized to the D1701-V Hin*dIII* fragment D* of 20.2 kbp in size (Figure 1B, Hin*dIII*). Similarly, the Eco*RI* fragments D (6.4 kbp) and E (4.17 kbp), which are both included in the Hin*dIII* fragment G of D1701-B (Figure 2), reacted with the enlarged fragment A* (ca. 64 kbp) and the shortened fragment D* (4.2 kbp) of D1701-V DNA (Figure 1B, Eco*RI*). To delineate the changed DNA fragments in more detail, many additional restriction enzyme digests and comparative Southern blot hybridizations were performed as well as cloning, subcloning and sequencing of those DNA fragments differing between D1701-B and D1701-V, respectively. All these numerous results are not individually presented because it would exceed by far the scope of this manuscript.

Finally, DNA sequencing of the cloned fragments differing between D1701-B and D1701-V allowed the accurate comparison and restriction mapping of the right-hand terminus of D1701-B DNA (comprising 36,525 bp) and of D1701-V (comprising 31,805 bp), as shown in Figure 2. As indicated by the dashed brackets in Figure 2C the D1701-V DNA revealed 2 deletions in the right-hand end of its genome compared to D1701-B (Figure 2B). The deleted part designated AT comprises 2,528 bp leading to the loss of the Hin*dIII* site between fragment I and J as well as the loss of the Eco*RI* site between fragment A and E of D1701-B (Figure 2B). Consequently, the DNA of D1701-V exhibits the shortened Hin*dIII* fragment I* and the missing Hin*dIII* fragment J as well as an enlarged Eco*RI* fragment A* (Figure 2C).

The second deleted part in D1701-V is designated D and comprises 2,195 bases and is located 7,920 bases to the right of locus AT, which resulted in the loss of another Hin*dIII* site between fragments G and D of D1701-B (Figure 2). Therefore, the Hin*dIII* fragment G is no more existent in D1701-V but the enlarged fragment D* (Figure 2C). Finally, a third deleted region named A was detected in the left-hand terminus of D1701-V (Figure 2D) and comprises 2,255 bases resulting in the truncated D1701-V fragments Hin*dIII* – C*, Eco*RI* – B* and Kpn*I* – G* (Figure 1 and Figure 2D).

### 3.2. ORFV Genes Affected by the 3 Deletions

**Figure 3:**
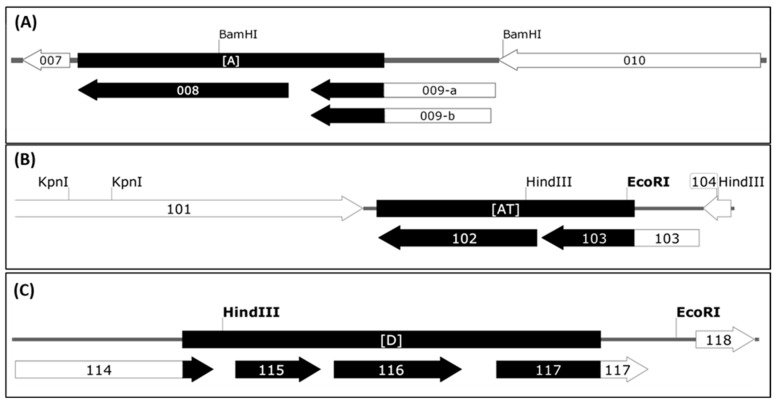
Open reading frames affected by the 3 deletions in D1701-V. The ORFs of the genomic regions inclosing deletions A (**A**), AT (**B**) and D (**C**) are depicted. The ORFV genes are numbered according to Delhon et al. [61], the deleted parts are shown with black boxes and black arrows. The gene maps were constructed using SnapGene Viewer software.

Sequence comparison of the genomic parts covering the deletions A, AT and D revealed those open reading frames (ORFs) of the D1701-B virus genome affected by the Vero cell culture adaption. As shown in Figure 3A, deletion A removed the complete ORF008 (1,551 bp) as well as 543 bp of the C-terminus of ORF009. Due to the presence of two in frame ATG start codons the resulting shortened gene ORF009 of D1701-V can theoretically comprise either 273 aa or 262 aa (Figure 3A, 009-a and 009-b) and terminates at a translational stop codon immediately upstream of locus A. The flanking ORF007 encoding the ORFV *dUTPase* [60] as well as the ORF010 were conserved.

The deletion AT (Figure S2) also affected 2 ORFV genes as depicted in Figure 3B. The complete ORF102 (1,557 bp in size) encoding the so-called ATI/fusion protein (519 aa) has been completely deleted and 912 bp (304 aa) of the C-terminus of ORF103 encoding the ATI gene (1,548 bp in size, 516 aa) are removed. Consequently, this deletion results in a new ATI variant composed of the original N-terminal 211 aa plus 31 new aa. The flanking essential ORFV genes, ORF101 encoding the RNA polymerase subunit RPO132 and ORF104 encoding the ORFV fusion protein (10 K gene) essential for the virion criss-cross structure [53] are completely retained.

The deletion D (Figure 3C, Figure S2) had occurred in the right terminal quarter of the viral genome, 7,920 nt downstream of locus AT. The deleted part eliminates 165 bp (54 aa) of the C-terminus of gene ORF114 (1,041 bp, 346 aa) and the successive genes ORF115 (447 bp, 149 aa) and ORF116 (672 bp, 224 aa), respectively. Additionally, 547 bp of the N-terminus of ORF117 were removed, which is an intermediate ORFV gene encoding the ORFV GIF gene, a GM-CSF-/IL2-inhibitory factor [62,63]. The deletion created a new potential ORF by fusing the N-terminal 291 aa of ORF114 with the C-terminal 83 aa of GIF. Whether this “114–117” ORF (1,125 bp, 374 aa) is expressed in D1701-V infected cells is not known. In sum, three regions of the D1701-B genome comprising in total almost 7 kbp were lost during multiple passaging in Vero cells that resulted in deletion of 4 complete ORFV genes as well as truncation of 4 other genes.

Recently a genomic sequence of a BK-KL3A cell propagated D1701 virus was reported by [64], which will be designated in the following D1701-McG for better discrimination. Sequence of the presented AT and D locus of D1701-B was compared with D1701-McG and other ORFV strains as summarized in Table 2 and Figure S2. DNA sequence identity of gene ORF102 was found between D1701-B and D1701-McG and high homology to that of the ORFV strains NZ2 and B029 but lower nt identity (85%) to OV-IA82. The ORF103 represented a similar relationship, although with only 65% nt identity to ORFV OV-IA82, whereas this gene is lacking in B029 (Table 2). The sequence comparison of the genes located in locus D and deleted in D1701-V revealed that the ORF114 exhibited close homology and identical size in all compared ORFV strains. In contrast, ORF115 exhibited only 88% or 89% nt identity and a smaller gene size when compared between D1701-B and D1701-McG and the other ORFV strains (Table 2). Similarly, the ORF116 of the 3 ORFV strains displayed size differences and nt identities between only 81 and 83% in comparison to D1701-B and D1701-McG. The GIF gene (ORF117) sequences revealed a close relationship among D1701-B, NZ2, OV-IA82 and B029 with respect to gene size, aa and nt identities. As outlined in Figure S3A, the GIF of D1701-B and D1701-McG differs in 9 single aa, the WSXWS motif important for biological function is unaltered. Surprisingly, the published sequence data of D1701-McG predict an enlarged ORF117 comprising 304 aa instead of 265 aa (Table 2, Figure S3A). This difference is ascribed to an additional A at nt position 724 in the D1701-McG sequence, which consequently leads to a frame-shift from aa 242 on and a more distantly used stop codon compared to the GIF genes of the other ORFV (Figure S3B). Finally, the not deleted ORF118 downstream of locus D in D1701-B demonstrated high sequence homology albeit differing gene sizes (Table 2). In B029 ORFV the sequence of the suspected truncated ORF118 could be not resolved unambiguously [65].

### 3.3. Use of Deletion D for Foreign Gene Expression

**Figure 4:**
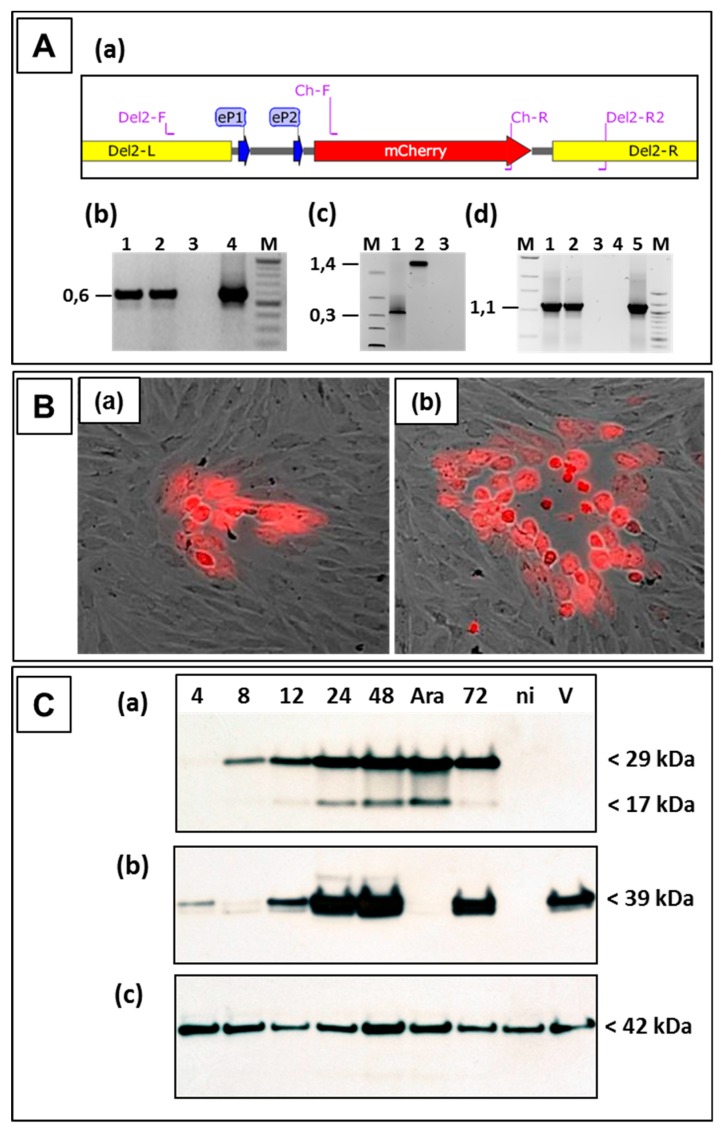
Legend: Characterization of the ORFV recombinant D1701-V-D12-mCherry. (**A**) PCR demonstrating correct transgene insertion. (**a**) Map section of plasmid pD12-mCherry illustrates location of the used primers also described in Table 1. The left (Del2-L) and the right (Del2-R) homology arm for recombination, early promoter eP1 and eP2 and the *mCherry* gene are shown. (**b**) Using primer pair Ch-FR the *mCherry*-specific amplicon of 0.59 kbp in size was obtained with DNA from red virus plaques (lanes 1 and 2) and with control plasmid pD12-Cherry (lane 4) but not in non-infected cells (lane 3). (**c**) Shows the result of the D locus-specific PCR Del2-FR2. Using DNA from D12-Cherry infected Vero cells (lane 2) the specific 1.4 kbp amplicon was obtained, whereas DNA from cells containing the parental D1701-V (lane 1) results in a 0.39 kbp amplicon due to the absence of gene insertion. DNA from non-infected cells (lane 3) was used as negative control. (**d**) Demonstrates the results of the PCR with the primers Del2-F and Ch-R. The specific amplicon of 1.1 kbp was obtained with DNA from D12-Cherry infected cells (lanes 1 and 2) or with plasmid pD12-mCherry (lane 5). DNA from non-infected cells (lane 3) and without template DNA (lane 4) were used as negative controls. In lanes M size marker (1 kbp ladder or 100 bp ladder, NEB) were separated. On the left the sizes are given in kbp. (**B**) Expression of *mCherry* allows identification of red fluorescent virus plaques of the ORFV recombinant D1701-V-D12-mCherry (D12-Cherry), which became clearly visible 12 h (a) and 24 h (b) after infection. Microscopic magnification 40×. (**C**) Western blot analysis of Vero cells infected with the recombinant D12-Cherry. (**a**) The mCherry was detectable with a specific antiserum during 4 to 72 h after infection but not in non-infected (lane ni) or in D1701-V infected (lane V) Vero cells. In infected cells treated with AraC to arrest ORFV gene expression in the early phase *mCherry* expression was detectable, too. Panel (**b**) demonstrates expression of the late major envelope protein (ORF059) with monoclonal antibody 4D6 in cells infected with the recombinant as well as with parental D1701-V but not in recombinant virus cells treated with AraC or in non-infected cells. (**c**) The protein load of each sample was tested using a specific antiserum against cellular β-actin. The sizes are indicated on the right.

The following experiments were performed to investigate the suitability of locus D as a novel insertion site for stable expression of foreign genes in ORFV D1701-V. After nucleofection of D1701-V-infected cells with pD12-mCherry numerous red fluorescent virus-infected cells could be selected by fluorescence microscopy. Red virus plaques were tested by PCR assays for the presence and correct insertion of *mCherry*. Using primer pair Ch-F and Ch-R (Figure 4Aa and Table 1) the specific amplicon of 0.59 kbp displayed the presence of *mCherry* in different red virus plaques (Figure 4Ab, lanes 1 and 2). The amplification of the 1.4 kbp product with primer pair Del2-F and Del2-R2 proved the insertion of the *mCherry* gene in locus D (Figure 4Ac, lane 2), whereas the empty D locus in the parental D1701-V resulted in amplification of only 0.39 kbp (Figure 4Ac, lane 1). PCR results with primers Del2-F and Cherry-R corroborated the precise insertion of *mCherry* into the D locus; primer location is shown in Figure 4Aa. The specific amplicon of 1.1 kbp was obtained with DNA isolated from D12-Cherry virus-infected cells (lanes 1 and 2) and with control plasmid pD12-mCherry (lane 5). DNA sequencing of these PCR products additionally assured the presence of the correctly inserted gene. After limiting dilution red virus plaques (Figure 4B) could be used for final plaque purification to obtain the homogenous recombinant virus D1701-V-D12-mCherry, abbreviated in the following as D12-Cherry. 

The mCherry protein expression in infected cells was demonstrated by Western blot analysis and flow cytometry. As shown in Figure 4C, mCherry expression (29 kDa) was detectable already 4–8 h after D12-Cherry virus infection increasing with later times. ORFV gene expression was arrested in the early phase by AraC treatment and showed strong early mCherry expression in D12-Cherry-infected cells (Figure 4Ca lane Ara). The protein of the smaller mol. mass (17 kDa) represents a processed form of mCherry. As expected mCherry was not detected in non-infected or parental D1701-V-infected cells (Figure 4Ca, lanes ni and V). The major envelope protein F1L of ORFV (gene ORF059) was detected with the specific monoclonal antibody 4D9 at later times of infection but as a late ORFV protein not in AraC-treated infected cells (Figure 4Cb). Finally, comparable protein load of the different virus lysates was tested by cellular β-actin expression (Figure 4Cc). Flow cytometry demonstrated that 48 hours after D12-Cherry infection (moi = 2.0) approximately 48% of the Vero cells were positive for mCherry. 

**Figure 5:**
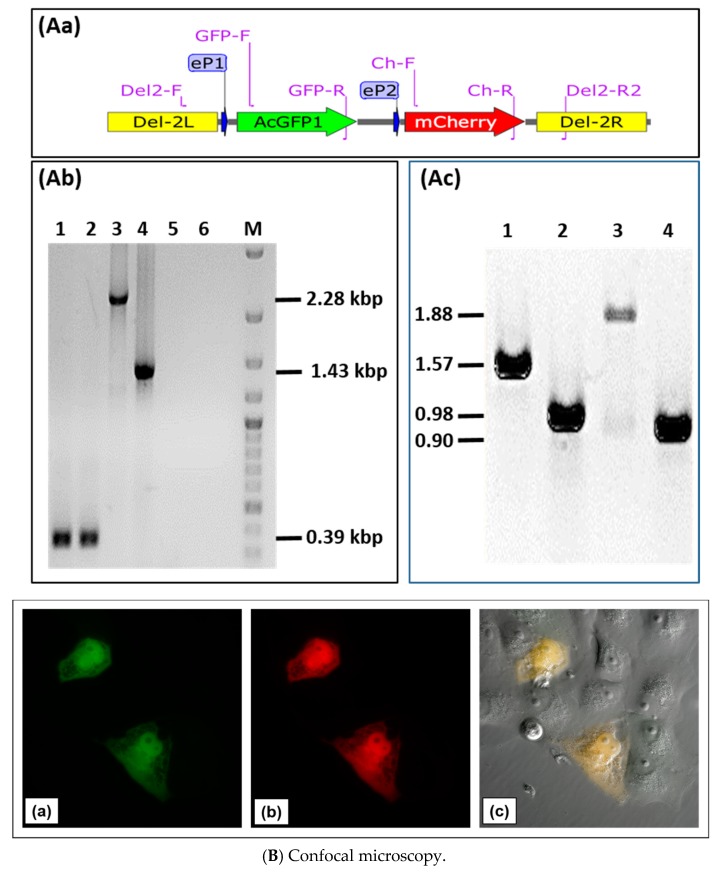

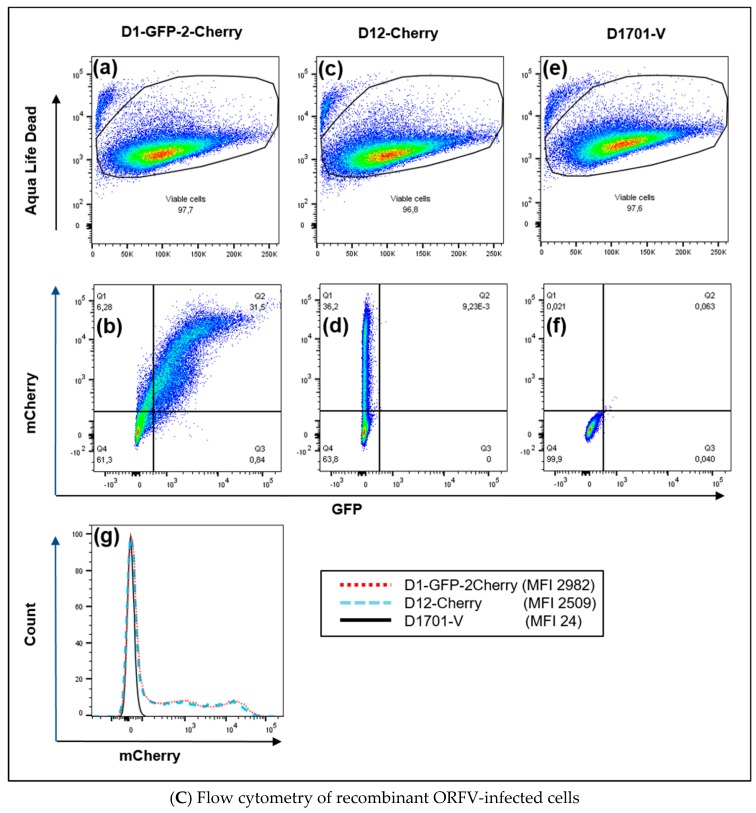
Characterization of ORFV recombinant D1701-V-D1-GFP-2-Cherry. (**A**) Insertion site- and insert-specific PCR. (**Aa**) Illustrates location of primers in plasmid pD1-AcGFP-2-mCherry, which are used for locus D-specific PCR (Del2-F and DEl2-R2), for mCherry- (Ch-F and Ch-R) and for AcGFP-specific PCR (GFP-F and GFP-R); eP1 and eP2 denote the early promoters. (**Ab**) The Del2-FR2 PCR with DNA from parental D1701-V infected Vero cells results in 0.39 kbp amplicon (lanes 1 and 2) due to the lack of gene insertion. After infection with the recombinant D1-GFP-2-Cherry the specific 2.28 kbp amplicon (lane 3) was obtained and with recombinant D12-Cherry the specific 1.43 kbp PCR product was amplified (lane 4). No specific amplification was found with DNA from non-infected cells (lane 5) and H2O as another control (lane 6). Lane M shows separation of size markers (1 kbp ladder, NEN Biolabs). (**Ac**) Correct insertion of *AcGFP* and *mCherry* gene in DNA of recombinant D1-GFP-2-Cherry was proven by PCR using the following primer pair combinations: GFP-F and Ch-R (lane 1, 1.57 kbp amplicon), Del2-F and GFP-R (lane 2, 0.98 kbp amplicon), GFP-F and Del2-R2 (lane 3, 1.88 kbp amplicon), Ch-F and Del-2R2 (lane 4, 0.90 kbp amplicon). (**B**) Confocal microscopy Vero cells were infected overnight with the recombinant D1-GFP-2-Cherry (moi 1.0), fixed and inspected for green fluorescence of AcGFP (**a**), red fluorescence of mCherry (**b**) and both merged (yellow) in combination with phase contrast microscopy (**c**). Microscopic magnification 40×. (**C**) Flow cytometry Viability (Aqua Life Dead) and fluorescent protein expression in Vero cells 24 hours after infection (moi of 2.0) with ORFV recombinant D1-GFP-2-Cherry (**a,b**), with ORFV recombinant D12-Cherry (**c,d**) and as a negative control with D1701-V (**e,f**). In (**b**), (**d**) and (**f**) the *X*-axis shows the number of GFP (FITC) -positive cells and the *Y*-axis the number of mCherry (PE)-positive cells. The overlay of the histograms of (**b**), (**d**) and (**f**) is plotted in (**g**) for direct comparison of mCherry-positive cells. The mean mCherry fluorescence intensity (MFI) is given in the inset.

Next, we tested the possibility of insertion and expression of two different transgenes in locus D of D1701-V. Plasmid pD1-AcGFP-D2-mCherry was constructed to allow expression of the two fluorescent marker genes *AcGFP* and *mCherry* in locus D. To this end, the Hind*III*-Spe*I* fragment of pAcGFP was inserted into plasmid pD12-mCherry to control *AcGFP* expression by promoter eP1, whereas *mCherry* expression remained regulated by promoter eP2 (Figure 5Aa). Vero cells were infected with the ORFV recombinant D12-Cherry and nucleofected with plasmid pD1-AcGFP-2-mCherry. After selection of candidates for the new recombinant D1701-V-D1-AcGFP-2-mCherry, abbreviated as D1-GFP-2-Cherry, PCR assays were used for identification. Insertion of both genes into locus D was demonstrated with the PCR primer combination Del2-F plus Del2-R2 (Figure 5Aa for primer location, Table 1). In case of the empty D1701-V this PCR led to the 0.39 kbp amplicon (Figure 5Ab, lanes 1 and 2) but after insertion of *AcGFP* and *mCherry* it produced a specific amplicon of 2.28 kbp (Figure 5Ab, lane 3), whereas the smaller 1.43 kbp PCR product was obtained with the parental virus D12-Cherry (Figure 5Ab, lane 4). No amplification was seen with both negative controls (Figure 5Ab, lanes 5 and 6). Figure 5Ac exemplifies the possibility of transgene-specific PCR using the following primer combinations: Lane 1 shows the 1.57 kbp product with primer GFP-F plus Ch-R comprising parts of the *AcGFP* and *mCherry* gene, lane 2 the 0.98 kbp amplicon with primer Del2-F plus GFP-R, lane 3 the 1.88 kbp product with primer GFP-F plus Del2-R2 and lane 4 amplification of 0.90 kbp with primer Ch-F plus Del2-R2. Together, the established PCR assays enable not only gene-specific but also insertion site-specific detection with DNA isolated from recombinant virus-infected cells. To get an idea on the sensitivity of the PCR tests, reconstruction experiments revealed a limit of detection of ca. 8 copies of D12 recombinant DNA per 100 cells using PCR Del2-FR2 and ca. 1 genome copy per 600 cells applying PCR Ch-FR.

Co-expression of *AcGFP* and *mCherry*, which are directed by the early promoter eP1 and eP2, respectively, could be illustrated in D1-GFP-2-Cherry infected cells by confocal microscopy. Cells exhibiting bright green fluorescence (Figure 5Ba) also expressed mCherry (Figure 5Bb), which can be seen by merging the green and red fluorescence (Figure 5c). The combined phase contrast photography also reveals surrounding non-infected cells. The successful intracellular expression of the fluorescent genes inserted into the D locus could also be demonstrated by flow cytometry.

Twenty-four hours after infection with approximately moi 2.0 of the recombinant D1-GFP-2-Cherry, 31.5% of almost 98% viable cells were found double positive for *AcGFP* and *mCherry* expression (Figure 5Ca,b-Q2). D12-Cherry infection resulted in only mCherry single positive cells (36.2%) and were negative for GFP (Figure 5Cc,d). As expected neither GFP- nor mCherry-positive cells were detectable after infection with ORFV D1701-V (Figure 5Ce,f). In all cases approximately 96% viable cells were obtained (Figure 5Ca,c,e). The histogram illustrates that almost identical numbers of mCherry-positive cells could be detected in both D1-GFP-2-Cherry- and D12-Cherry-infected cells (Figure 5Cg). Two mCherry-positive cell populations appeared after infection with both *mCherry* containing recombinants differing in the amount of expressed mCherry (Figure 5Cg). This can be explained by the presence of cells containing differing amounts of mCherry produced, since the infectious cycle was not synchronized.

**Figure 6:**
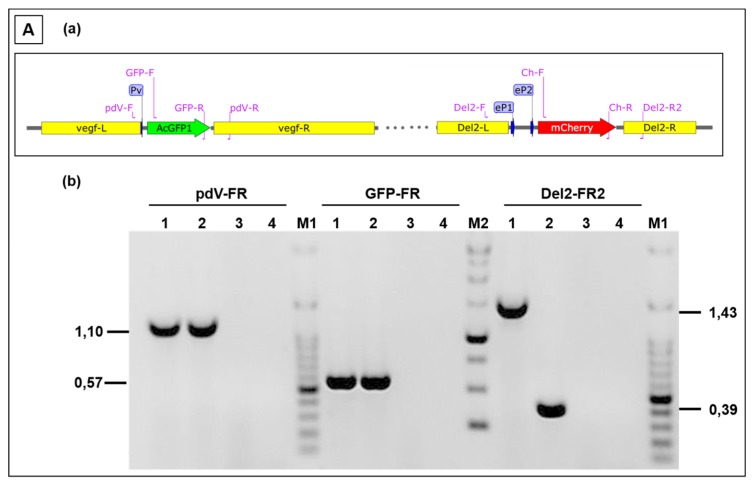

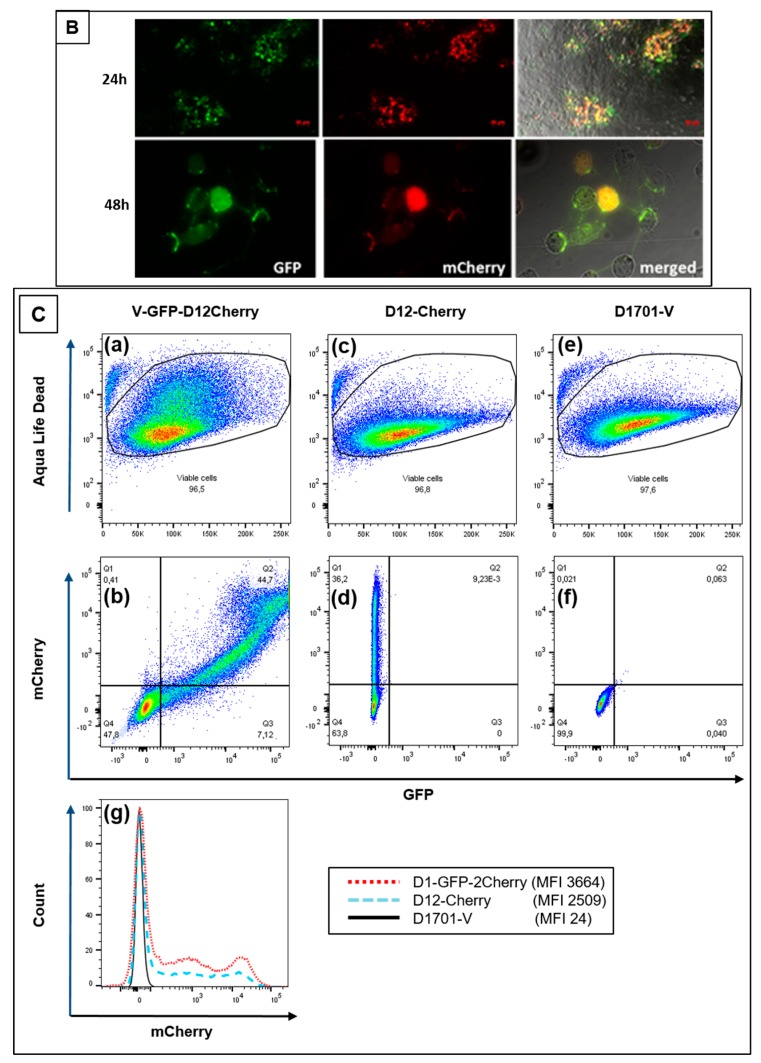
(**A**) PCR to demonstrate correct insertion of *AcGFP* in the V locus and *mCherry* in locus D. (**a**) The map location of primers is depicted, the homology arms vegf-L and vegf-R as well as Del2-F and Del2-R, the early promoters Pv, ep1 and eP2 are schematically drawn. (**b**) The results of the PCR with the indicated primer combinations are separated in 0.8% agarose gels with templates isolated from (**lanes 1**) V-GFP-D12-Cherry infected cells, (**lanes 2**) V-gfp infected cells, (**lanes 3**) non-infected cells and (**lanes 4**) non-template reaction. The amplicon sizes are indicated in kbp, DNA size markers are separated in lanes M1 (100 bp ladder) and lane M2 (1 kbp ladder). (**B**) Confocal images showing plaque formation of Vero cells 24 and 48 hours after infection with moi 2.0 of D1701-V-AcGFP-D12-mCherry (V-GFP-D12-Cherry). Concurrent expression of *AcGFP* (green) and *mCherry* (red) are demonstrated in each infected cell (merged). Microscopic magnification 10× (24 h) and 20× (48 h). (**C**) Flow cytometric determination of viability (Aqua Life Dead) and fluorescent protein expression 24 hours after infection (moi of 2.0) with V-GFP-D12-Cherry (**a,b**), with D12-Cherry (**c,d**) and as a negative control with D1701-V (**e,f**). The dot plots (b), (d) and (f) show on *X*-axis the number of AcGFP-positive cells and on Y-axis the number of mCherry-positive cells. The overlay of the mCherry histograms of (b), (d) and (f) is plotted in (**g**) for direct comparing the number of mCherry-positive cells; the mCherry mean fluorescence intensity (MFI) is given for each virus-infected cells in the inset.

Finally, the simultaneous expression of foreign genes in the 2 different insertion loci V and D of D1701-V, respectively, was also achieved. Plasmid pV-AcGFP, expressing the *AcGFP* gene under the control of Pv, was used for nucleofection of cells infected with the above described recombinant D12-Cherry. Selecting for *mCherry* and *AcGFP* double-positive cells finally resulted in purification of the recombinant D1701-V-AcGFP-D12-mCherry (abbreviated V-GFP-D12-Cherry). Figure 6A demonstrates PCR results verifying correct integration of the *AcGFP* gene in the V locus and of the *mCherry* gene regulated by eP2 in the D locus. Amplicons of the expected size were obtained with template DNA isolated from V-GFP-D12-Cherry-infected cells (Figure 6Ab, lanes 1) after *vegf-e* locus specific PCR pdV-FR (1,108 bp), AcGFP-specific PCR GFP-FR (574 bp) and D locus-specific PCR (1,435 bp). DNA isolated from cells infected with virus V-GFP yielded the expected AcGFP-specific amplicon and due to the empty D locus the Del2-FR amplicon was only of 388 bp in size (Figure 6Ab, lanes 2). The negative control included DNA from D1701-V-infected cells (lanes 3) and H_2_O as non-template control (lanes 4). Figure 6B shows concurrent green (AcGFP) and red (mCherry) fluorescence in identical virus plaques or single cells (merged) after 24 or 48 h.p.i. Flow cytometry revealed that 24 h after infection with V-GFP-D12-Cherry (moi 2.0) almost half of the infected cells expressed both *AcGFP* and *mCherry*, respectively (Figure 6Cb-Q2). After infection with the single recombinant D12-Cherry 36% mCherry-positive cells were measured (Figure 6Cd-Q1) and as expected control infection with D1701-V remained negative both for mCherry and AcGFP (Figure 6Cf). A direct comparison of mCherry-positive after infection with each virus is given in Figure 6Cg. The histogram overlay again reveals 2 cell populations positive for mCherry as also observed after infection with other recombinants (see above). Taken together, the presented experiments demonstrated that several foreign genes can be simultaneously early expressed not only in locus D or V but also in both loci. 

### 3.4. Evaluation of Expression Strength Dictated by the used ORFV Early Promoters

Knowledge of the magnitude of expression strength of genes regulated by the 3 used early promoters can be important for the generation of transgene expressing ORFV recombinants. Thus, we quantified the expression of the fluorescence marker genes *AcGFP* and *mCherry*, respectively, regulated by each of the early promoters Pv, eP1 and eP2. The marker gene expression in the infected cells was measured by flow cytometry and was used as a readout for promoter strength. The performed experiments allowed us to examine an influence of the integration of not only one foreign gene in one or both insertion loci of the viral genome (V, D) but also whether the use of each of the 3 early promoters either in the V or in the D locus might exert different effects on the expression strength of the controlled foreign genes. For a valid comparison of the results similar infection rates of each recombinant were tested after 20 h.

**Figure 7:**
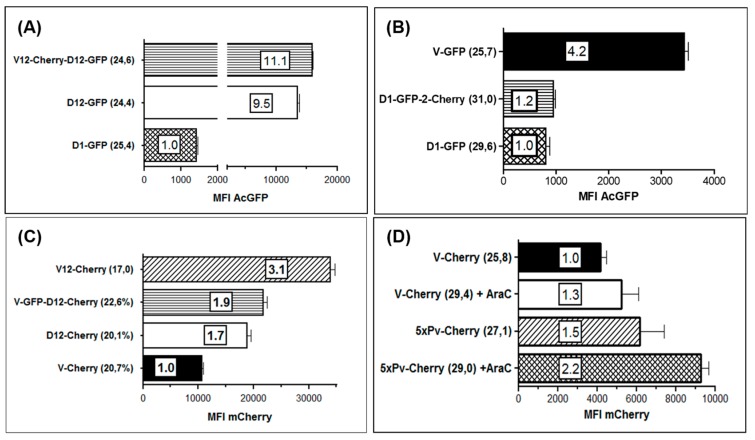
Comparison of strength of the promoters Pv, eP1 and eP2 by flow cytometry. Vero cells were infected in triplicates with the indicated ORFV recombinants for 22 h and the mean fluorescence intensity (MFI) was measured by flow cytometry. The numbers in parentheses behind the name of each recombinant indicate the percentage of positive cells determined by flow cytometry. The numbers in the bars denote the multiple of the particular lowest MFI set to 1.0. (**A**) Activities of eP1 and eP2 in insertion site D directing expression of *AcGFP* gene. The lowest MFI was found with promoter eP1 (recombinant D1-GFP) and set to 1.0, whereas expression from eP2 was 11.1- or 9.5-fold stronger after infection with V12-Chery-D12-GFP or D12-GFP, respectively. (**B**) Again, the lowest promoter activity was demonstrated from eP1 (recombinant D1-GFP and D1-GFP-2-Cherry) but 4.2-fold higher expression strength by promoter PV. (**C**) Expression of *mCherry* in insertion site V under control of Pv (recombinant V-Cherry) yielded the lowest MFI (set to 1.0) but was 3.1-fold higher when controlled by eP2 (recombinant V12-Cherry). Expression in the D locus showed that the eP2 led to 1.9 higher (recombinant V-GFP-D12-Cherry) or 1.7 higher (recombinant D12-Cherry) MFI. (**D**) The effect of a tandem arrangement of 5 core elements of Pv (recombinant 5xPv-Cherry) on the expression of *mCherry* in the V locus was compared to that of the single Pv (V-Cherry; set to 1.0). Vero cells were infected in the absence or in the presence of AraC (+ AraC).

A comparison of the promoter activities in the D locus revealed an approximately 10-fold higher expression of AcGFP from eP2 (Figure 7A, D12-GFP) as compared to eP1 (Figure 7A, D1-GFP). The high activity of eP2 did not substantially change when a second foreign gene (*mCherry*) was expressed for instance in the V locus (Figure 7A, V12-Cherry-D12-GFP). Similarly, additional gene expression in locus D controlled by eP1 did also not alter or influence the strong gene expression from eP2 (data not shown). The highest activity of eP2 promoter was found in the V locus (Figure 7C, V12-Cherry). Here it gave rise to a threefold higher foreign gene expression as compared to the original promoter Pv, whereas eP2 in the D locus displayed an almost 2-fold higher activity than Pv (Figure 7B). A comparable high potency was never observed with eP1 neither in the V nor in the D locus and also not in cells exhibiting higher infection rates.

Recently it was reported that tandem repetition of the core of the early poxviral promoter led to increased foreign gene expression in VACV vector MVA, which was accompanied by improved booster immunization and increased antigen-specific memory T-cell response [21,57,66]. Thus, we compared the *mCherry* expression controlled by Pv (virus V-Cherry) with that regulated by promoter 5xPv, which contains 5 tandemly repeated Pv core elements (virus 5xPv-cherry). To inhibit ORFV replication and late gene expression, the infected Vero cells were additionally treated with AraC. It can be seen in Figure 7C and 7D that *mCherry* gene expression was equally detectable in the presence or in the absence of AraC, as expected for the activity of a true early ORFV promoter. The tandem repetition of five Pv core elements at most duplicated the mCherry MFI (Figure 7D). A very similar small boosting effect was observed by the reiteration of five eP2 core elements downstream of Pv as compared to gene regulation by Pv or eP2 alone. Conclusively, successive repetition of the core elements of Pv or eP2 can slightly increase the foreign gene expression in ORFV, however, for future gene constructs the use of the early promoter Pv or eP2 will be sufficient.

## 4. Discussion

The ORFV strain D1701, originally isolated from a diseased sheep, was attenuated by multiple passages in primary ovine culture cells [21]. Already since the nineties the virus resulting from propagation in BK cells (referred to as D1701-B) was used for the production of the commercially supplied inactivated form as an unspecific immune modulator registered as Baypamun or Zylexis (for review [27,67]). Before discussing the presented results, we believe it imperative to emphasise that exactly that D1701-B virus was formerly also subject for our published D1701 genome analyses [18,28,54] and was used here for Vero cell adaptation. This is important to be aware of by assessing the DNA sequence data of a D1701 virus published and deposited at GenBank [64]. The not sequenced genomic termini do not allow identification of the ITR, which should be typically enlarged in D1701 due to the reported genetic transpositions and deletions [28]. Sequencing of the complete genome of our different available D1701 variants is underway and will be subject of an upcoming publication. For the generation of ORFV recombinants the feasible use of Vero cells instead of BK-KL3A cells clearly facilitated the transfection or nucleofection. Moreover, the presented results are of importance for the ORFV vector platform based on the D1701-V virus, because not only the data now exactly delineate the genomic alterations that had occurred after adapting the D1701-B virus to grow in Vero cells but also they demonstrate the additional utility of locus D for foreign gene expression. Enhancing the capacity of simultaneous expression of different transgenes certainly broadens the potential of the ORFV vector platform.

**Figure 8:**
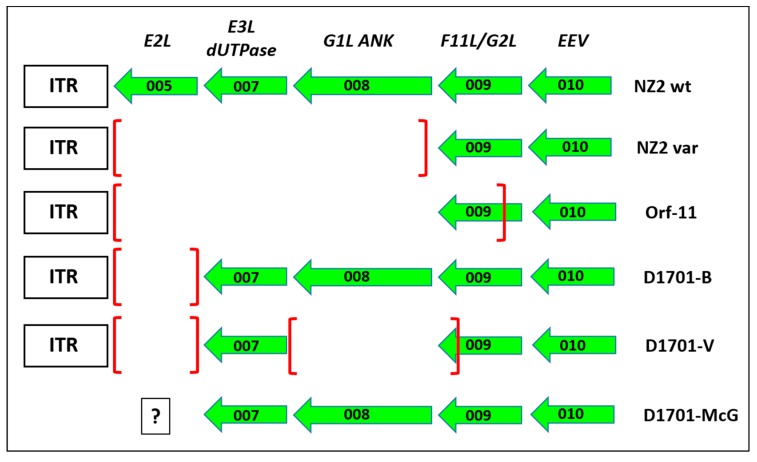
Comparison of gene deletions in the left hand end of ORFV genomes The gene deletions mapped in the left terminus of the genome of the different cell culture passaged ORFV variants NZ2var [72] and Orf-11 [73] are depicted in comparison to the prototype ORFV wild-type (wt) strain NZ2 and to the D1701 derivatives as explained in the text. The earlier first designations for the ORFV genes are given in the upper line, EEV means that this ORF010 encodes an ORFV envelope protein. ITR denotes the inverted terminal region. The framed question mark given for D1701 virus sequenced by McGuire (D1701-McG) indicates the lack of sequence data left to the ORF007. The squared brackets mark the deleted part in each virus variant.

Compared to D1701-B the adaption to grow in Vero cells created three additional major deletions. At the left-hand end of the genome, in the part called locus A, the ORF008 encoding Ankyrin repeat containing poxviral F-box protein [68,69,70] and the C-terminus of gene ORF009 is lost in strain D1701-V (Figure 8). These genes are retained in D1701-B [28] as also in other DNA sequenced *Parapoxviruses*, while ORF008 was reported to differ between ORFV and BPSV [71]. 

The latter ORFV gene product can in vitro bind to and sequester the cellular factor FIH from the cytoplasm to the nucleus and prevent inhibition of the hypoxia-inducible factor HIF and a role in viral virulence might be possible [74]. The function of ORF009, earlier named G2L [70], is not known though it displays some homology with the VACV F11L, which is involved in virus release and efficiency of virus spread by inhibition of cellular RhoA signalling [75]. The truncated ORF009 of D1701-V displays two potential encoding ORF, because 2 in frame ATG start codons are created (Figure 3A: ORF 009-a and ORF 009-b, respectively). BLAST search reveals that the sequence of some ORFV strains (like OV-IA82, SA00 or B029) also contains these 2 start codons, whereas other ORFV strains (like NZ2) exhibit only the second protein start codon. Other cell culture-passaged, attenuated ORFV variants (Nz2var and Orf-11) have lost the left 3 genes during tissue propagation and Orf-11 lost almost 80% of ORF009 (Figure 8). Since both cell culture-adapted strains exhibit clearly reduced virulence, these dispensable genes were assumed to be associated with ORFV attenuation [72,73]. The possibility of using ORF008 for foreign gene insertion was described earlier with ORFV strain NZ2 by exchanging it for an *Echinococcus* antigen [76]. However, a protective potency of this ORFV recombinant has not been provided. The usefulness of locus A in D1701-V for insertion and expression of foreign genes remains to be analysed in more detail. The suitability of this locus for the generation of stable ORFV recombinants could be problematic due to the reported high variability of the left terminus of the *Parapoxvirus* genome [72,73,77,78]. On the other hand, after more than 20 additional passages of D1701-V in Vero cells or in BK cells we could not detect further deletions at the left genomic terminus. Interestingly the *dUTPase* encoding ORF007 remained preserved in D1701. The presence or absence of ORF005 in D1701-McG (D1701 virus published by McGuire et al.) cannot be determined due to the lack of available sequence data.

The next deleted part, locus AT, is located in the rightward third of the genome of D1701 (Figure 3 and Figure S2). The complete ORF102 and the C-terminal half of ORF103 was eliminated, both genes dispensable for ORFV D1701-V. Their homology to VACV genes predicts an involvement in virus-filled A-type inclusion bodies (ATIs), which, however, do not exist in ORFV. Among the *Parapoxviruses* they are both reported as genes of unknown function with marked sequence variation and remarkable low G+C content [61,65,77,79]. D1701-V is the first ORFV variant that demonstrates a deletion affecting these two genes. The flanking essential genes ORF101 (RNA polymerase subunit 132) and ORF104, which encodes the ORFV fusion protein essential for the virion crisscross structure [53], are both preserved in D1701-V. Preliminary data indicate the possibility to generate recombinants with foreign genes integrated in the AT locus but more studies for their stability are still needed.

The deletion D comprises 2,195 base pairs in the right terminal quarter of the D1701-V genome ensuing the destruction of 4 genes, which thus, are also non-essential for virus replication. The C-terminus of ORF114 is truncated and the following genes ORF115 and ORF116 are eliminated. Again, so far no functions can be attributed to any of these ORFV genes. The variability of this genomic part of *Parapoxviruses* is reported also by others including different deletions or indels [65,71,79,80,81]. Due to the observation of almost equal length of non-coding and coding fragments it is speculated that in earlier phases of ORFV evolution some of these genes might have been part of coding regions [61]. A similar deletion was also found in PCPV (*Pseudocowpoxvirus*) isolates after in vitro culturing [80]. The N-terminus of the proximate gene ORF117 is deleted after adaption to Vero cell growth. This intermediate-late expressed gene was identified by Deane et al. [62] as the ORFV-encoded GM-CSF/IL-2 inhibiting factor (GIF). In solution it forms functional dimers or tetramers and binds to ovine but not to human GM-CSF and IL2. Recently it was shown that the overlapping binding sites for GM-CSF and IL-2 are mutually exclusive [82]. In D1701-V the deleted N-terminal part of GIF removes residues crucial for the GIF binding activity comprising 2 glycosylation sites, the E-F loop and the WSXWS motif [63]. Therefore, we suppose that the D1701-V is devoid of a functional GIF, whereas the 9 single aa changes in GIF of D1701-B should not affect its biological function. GM-CSF as well as IL-2 play central roles in the recruitment, the differentiation and function of different immune and antigen-presenting cells (for review [15]). Since GIF is one of the ORFV genes suspected to subvert and to modulate the immune response (for review [16]), it is tempting to assume that the lack of a functional GIF gene might be one reason for the loss of virulence of D1701-V. Moreover, identified deletions in D locus may have a potential impact on immune regulatory activities of ORFV D1701-V, especially in the context of vaccine development. However, this affords additional studies and must be proven for example, by reconstitution of the deleted gene(s). Due to the DNA loss the N-terminal 165 nt of ORF114 are fused with the C-terminal 251 nt of GIF. Whether this newly created “114–117” hybrid ORF is expressed in D1701-V-infected cells remains to be investigated. Recently, two goat ORFV isolates were described possessing similar large DNA deletions that encompass 7 genes from ORF114 to ORF119 or the genes from ORF114 to ORF117. These variants of reduced virulence were obtained already after 3 culture passages [71].

Taken together the data indicate that especially this genomic region (locus D) might represent a kind of hot spot for the creation of gene variations and deletions not only in ORFV but also in other *Parapoxviruses*. Such a site for preferred loss of genetic material was reported for VACV at the right terminal inverted repeat boundary [83]. It is of note that the described deletions occurred only after repeated passaging in Vero cells but not after multiple passages in the originally used ruminant BK cells. Whether the loss of genetic material is associated with this monkey kidney cell line, which initially did not support productive replication of D1701, remains speculative as long as ORFV behaviour in other non-permissive cell lines has not been examined. Changed replication capacity, plaque or particle morphology or immunogenicity are not observed after adapting the D1701-B strain to Vero cell growth. Also, no further detectable gene deletions occurred after additional 10–20 Vero cell passages of the plaque-purified D1701-V. Hussain and Burger [17] investigated 3 ORFV isolates adapted to replicate in Vero cells after 4–6 cell culture passages. However, no data about the virological or genetic properties of the original virus isolates before growth in Vero cells were reported. A major advantage of the propagation of ORFV vector virus and its derivatives in Vero cells is the acceptance of this cell line for vaccine production. For instance, a well-characterized WHO Vero cell bank exists which is accepted by regulatory authorities for the production of human vaccines (for review see [5,84]).

The genomic changes accompanied by attenuation from D1701-B to D1701-V recall the derivation to the VACV vector virus MVA, which was also obtained after multiple passages in primary chicken cell cultures leading to the loss of approximately 15% of genomic information [85]. As MVA also the ORFV vector D1701-V belongs to risk group 1 according to its high attenuation profile. The potency of ORFV in non-permissive hosts offering a safety advantage in vaccine application when compared to the natural broad spectrum of *Orthopoxvirus* species. For selecting the new D1701-V recombinants by fluorescent marker gene swapping we used a comparable strategy reported for the production of single gene ORFV deletion mutants [49,86]. Starting with the different marker gene containing recombinants the relatively easy substitution or exchange of the fluorescent marker genes by any other gene of interest enables multiple transgene integration in the described insertion loci. The generation of the different ORFV recombinants was substantially accelerated by a flow cytometric pre-selection [52] in combination with the limiting dilution cloning. The stability of the inserted genes in both the D and the V locus has been shown during at least 10 cell culture passages. 

The efficient expression of various transgenes in D1701-V requires the use of a separate promoter for each inserted foreign gene. To this end, we wanted to keep our original concept of early transgene expression and were interested in additional new early promoters. The motifs of early promoters of ORFV and VACV are very similar [87] though the requirements of promoter sequences for maximal transcription can be different [88]. The novel synthetic ORFV early promoter eP1 and eP2, respectively, were designed on the basis of consensus motifs known from ORFV and VACV [7,44,45,88,89]. Surprisingly, the comparison of the expression strength of the fluorescent marker genes in cells infected with the different D1701-V recombinants revealed that the original ORFV*—gf-e* promoter Pv as well as the synthetic promoter eP2 display an up to 11-fold stronger expression than achieved with the promoter eP1, irrespective of the insertion site. The reason for that difference is obscure. As shown in Supplementary Figure S4, the only difference between promoter eP1 and both Pv and eP2 exhibits base 11. The weaker promoter eP1 contains at this position an A but eP2 and Pv a T. However, according to Davison and Moss [56] this residue is one of those with less or no effect on expression strength when changed. This observation might indicate that the characteristics of vaccinia and ORFV early promoters are not fully applicable. However, it needs further analyses of ORFV promoter sequence requirements. Influence of the sequence proximity of the promoter core on promoter strength differences [7] is also unlikely because it is almost identical. The distance between promoter core and gene, which was found to positively influence expression strength [89], comprises 32–35 nt for eP2 and 56 nt for both Pv or eP1 and therefore, seems also implausible. Finally, the distance of the early stop motif T5NT (U5NU in mRNA) from the end of the inserted gene also does not explain the difference in expression strength. Recently, it was reported that tandem repetition of the early promoter core can improve early foreign gene expression in VACV vector MVA, which was correlated with the magnitude of an antigen-specific immune response including enhanced CD8 T-cell and memory T-cell response [57,66,90]. In the presented experiments, however, the successive repetition of five core motifs of Pv or eP2 only slightly enhanced early foreign gene expression in the D1701-V vector. The presented experiments demonstrate that the possible integration and expression of a second foreign gene regulated by a separate early promoter does not negatively influence the expression of the first gene, independent from the insertion locus. Moreover, preliminary data indicate the possibility of expressing at least 3 transgenes in each tested insertion locus. Finally, when a lower foreign gene expression is needed in ORFV recombinants the promoter eP1 can be chosen, whereas stronger foreign gene expression is maintained by Pv or eP2, respectively.

## 5. Conclusions and Future Perspectives

The adaption of ORFV D1701 for growth in Vero cells is accompanied by loss of major genomic parts. After determination of their exact limits one of the deleted regions, locus D, is successfully used for integration and expression of foreign genes. For the first time ORFV recombinants are generated simultaneously expressing transgenes in two different insertion loci regulated by newly designed ORFV early promoters. These findings open new possibilities for the production of polyvalent ORFV vaccines combining several antigens or antigenic epitopes against one or against different pathogens. Moreover, it allows the combination of for example, distinct immunogens with immune enhancing or modulating factors in a single vectored ORFV vaccine.

## Figures and Tables

**Table 1:** Primer and promoter sequences. The critical core sequences of the synthetic early promoter eP1 and eP2, respectively, are given; the 3 consecutive early stop motifs T5NT in the Spacer region are underlined. The 16 nt core of early promoters is double underlined, the early stop motif T5NT is underlined. Sequences of the PCR primers used are listed.

Promoter eP1	5′-AAAAAAAAAATTGAAAAATTATTCTAAATATTGCACGG-3′
Promoter eP2	5′-AAAAATTGAAATTCTAACTTGTGTTCTTATAAATGAT-3′
Spacer SP	5′-TTTTTATCTTGTTTTTATCCTGTCTTTTTATCAGTTTTTTA GCTAGTTAAAACATAAATAGTAAAGCTAAAAAGAGACTATATCGGCGGCTGGAGTCTTGCAACAACCAGC-3′
Ch-F	5′-GCTTCAAGGTGCACATGGAGGGC-3′
Ch-R	5′-GGTGTAGTCCTCGTTGTGGGAGG-3′
GFP-F	5′-CCACAAGTTCAGCGTGAGCGGCGAG-3′
GFP-R	5′-GCGCTTCTCGTTGGGGTCCTTGGAC-3′
Del2-F	5′-GCAACAAGGTCTGCGTGCCTGCCGACC-3′
Del2-R2	5′-CGGCGATCTGGATGGTTGGAGCCATGCC-3′
pdV-F	5′-GGTGACGGTGCTCAGCGTGGTGGCGGTTTC-3′
pdV-R	5′-CTAGCGGCGTCTTCTGGGCGGCCTTGTGGT-3′

**Table 2:** Sequence comparison of genes of different ORFV strains located in the deletion loci AT and D. Sequence obtained from GenBank accession number for D1701-McG HM133903.1, for NZ2 DQ184476.1, for OV-IA82 AY386263.1 and for B029 KF837136.1.

Open reading frame (ORF)																								
	101			102			103			114			115			116			117			118		
ORFV strain	Length		Identity ^a)^	Length		Identity	Length		Identity	Length		Identity	Length		Identity	Length		Identity	Length		Identity	Length		Identity
	nt	aa	nt	nt	aa	nt	nt	aa	nt	nt	aa	nt	nt	aa	nt	nt	aa	nt	nt	aa	nt	nt	aa	nt
D1701-V	3483	1161	100%	―	―	―	―	―	―	―	―	―	―	―	―	―	―	―	―	―	―	306	102	100%
D1701-B	3483	1161	100%	1557	519	100%	1548	516	100%	1038	346	100%	447	148	100%	672	224	100%	795	265	100%	306	102	100%
D1701-McG	3483	1161	99%	1557	519	100%	1548	516	99%	1038	346	99%	447	148	100%	672	224	99%	912	304	99%	306	102	100%
NZ2	3483	1161	99%	1560	520	95%	1548	516	98%	1038	346	96%	435	145	88%	693	231	81%	795	265	96%	357	119	97%
OV-IA82	3483	1161	99%	1556	518	85%	1566	522	65%	1038	346	96%	429	143	89%	702	234	81%	795	265	96%	663	221	97%
B029	3483	1161	99%	1560	520	95%	―	―	―	1038	346	97%	435	145	89%	669	223	83%	795	265	96%	―	―	―

^a)^ Identity to D1701-B; nt = nucleotides, aa = amino acids; dash indicates deletion of the ORF.

## Patent

R.A. and H.-J.R. patent application WO2017/013023 A1.

## Supplementary Materials

The following are available online at http://www.mdpi.com/1999-4915/11/2/127/s1, Figure S1: Construction of plasmid pD12-mCherry, Figure S2: DNA sequence comparison of loci AT and D of the ORFV D1701 variants, Figure S3: Amino acid comparison of ORF117 (GIF) of different ORFV strains, Figure S4: Nucleotide sequence comparison of the core motifs of different poxviral early promoters.
