# Supplementary material for: Genomic Characterization of Orf Virus Strain D1701-V (Parapoxvirus) and Development of Novel Sites for Multiple Transgene Expression
Source: Viruses. 2019 Jan 30;11(2):127. doi: 10.3390/v11020127 (PMC6409557; doi:10.3390/v11020127)
Supplement: Supplementary file 1 [file viruses-11-00127-s001.pdf]

Supplementary Figure S1. Construction of plasmid pD12-mCherry

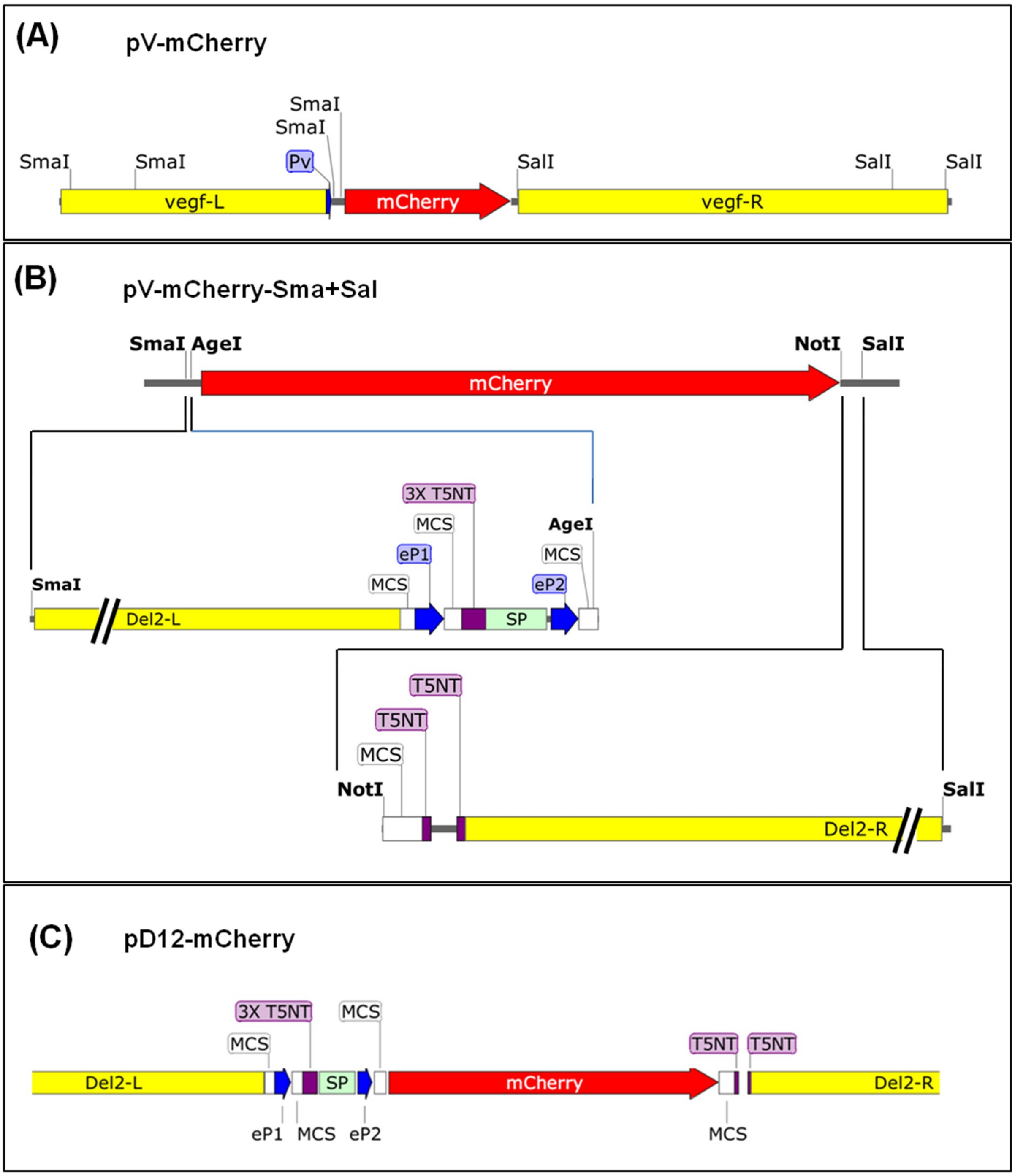

Legend to Figure S1: Construction of plasmid pD12-mCherry

(A) The *Sma*I and *Sal*I restriction sites in plasmid pV-mCherry are shown, which are necessary for the following plasmid construction. Left and right vegf-e gene homology

arms vegf-L and vegf-R and the promoter Pv are indicated.

(B) Re-ligation after restriction digest led to plasmid pV-mCherry-Sma+SaI, and using its indicated singular restriction sites, the left Del2-L as well as the right Del2-R arms were inserted as described in the text.

(C) Shows the obtained plasmid pD12-mCherry. The multiple cloning sites MCS, the early stop motif T5NT and the early promoter eP1 separated by spacer SP (see also Table 1) from early promoter eP2 are displayed.

Supplementary Figure S2. DNA sequence comparison of loci AT and D of the ORFV D1701 variants

```
          3601                                              3650
D1701-B   GACTTCTAGC TTCTTAGACC GATGCTACCA TATCGCGGCG TGCCGGCCCC
D1701-V   GACTTCTAGC TTCTTA.... ... Deletion AT ..... ..........
D1701-McG GACTTCTAGC TTCTTAGACC GATGCTACCA TATCGCGGCG TGCCGGCCCC

          6101                                              6150
D1701-B   TTGAACTTAT CAAACGAAAT GTTTACTCGC GGTTGTGCTT CGTATTTTTT
D1701-V   .......... .......... .......... .......... ..TATTTTTT
D1701-McG TTGAACTTAT CAAACGAAAT GTTTACTCGC GGTTGTGCTT CGTATTTTTT
```

```
          14051                                            14100
D1701-B   CAAGACGGAG CCGGCGCGGC GCAGCACGTC CTCGCCGGGC CCTAGACGCC
D1701-V   CAAGACGGAG CCGGCG.... ..... Deletion D .... ..........
D1701-McG CAAAACGGAG CCGGCGCGGC GCAACACGTC CTCGCCGGGC CCTAGACGCC

          16251                                            16300
D1701-B   CCAGCACGTT CTACGGCCAA ACCGCCGGTT CGCTCCAGCG AAGCTGCACA
D1701-V   .......... CTACGGCCAA ACCGCCGGTT CGCTCCAGCG AAGCTGCACA
D1701-McG CCAGCACGTT CTACGGCCAA ACCGCCGGTT CGCTCCAGCG AAGCTGCACA
```

Legend S2:

The DNA parts covering deletion site AT and D, respectively, were aligned by CLUSTAL Omega. Dashes indicate the deleted parts, stars mark identical nt.

Supplementary Figure S3A. Amino acid comparison of ORF117 (GIF) of different ORFV strains

```
              1                     ▼        ▼                      50
D1701-B       MACLRVFLAV LALCGSVHSA RWIGERDFCM AHAQDVFARL QVWMRIDRNV
D1701-McG     MACLRVFLAV LALCGSVHSA RWIGERDFCM AHAQDVFARL QVWMRIDRNV
NZ2           MACLRVFLAV LALCGSVHSA QWIGERDFCT AHAQDVFARL QVWMRIDRNV
OV-IA82       MACLRVFLAV LALCGSVHSA QWIGERDFCT AHAQDVFARL QVWMRIDRNV
B029          MACLRVFLAV LALCGSVHSA QWIGERDFCT AHAQDVFARL QVWMRIDRNV
                                    :        o

              51                     ▼                             100
D1701-B       TAADNSSACA LAIETPPSNF DVDVYVAAAG INVSVSAINC GFFNMRQVET
D1701-McG     TAADNSSACA LAIETPPSNF DVDVYVAAAG INVSVSAINC GFFNMRQVET
NZ2           TAADNSSACA LAIETPPSNF DADVYVAAAG INVSVSAINC GFFNMRQVET
OV-IA82       TAADNSSACA LAIETPPSNF DADVYVAAAG INVSVSAINC GFFNMRQVET
B029          TAADNSSACA LAIETPPSNF DADVYVAAAG INVSVAAINC GFFNMRQVET
                                     .              :

              101                    ▼          ▼                  150
D1701-B       TYNTARRQMY VYMDTWDPWV LNDPQPLFSQ EHENETLPYL LEVLELARLY
D1701-McG     TYNTARRQMY VYMDTWDPWV LNDPQPLFSQ EHENETLPYL LEVLELARLY
NZ2           TYNTARRQMY VYMDSWDPWV IDDPQPLFSQ EYENETLPYL LEVLELARLY
OV-IA82       TYNTARRQMY VYMDSWDPWM LDDPQPLFSQ EYENETLPYL LEVLELARLY
B029          TYNTARRQMY VYMDSWDPWV IDDPQPLFSQ EYENETLPYL LEVLELARLY
                                  : ::          :

              151                ▼                                 200
D1701-B       IRVGCTVPGE QPFEVIPGTD YPHTGMEFLQ HVLRPNRRFA PAKLHMDLEV
D1701-McG     IRVGCTVPGE QPFEVIPGTD YPHTGMEFLQ HVLRPNRRFA PAKLHMDLEV
NZ2           IRVGCTVPGE QPFEVIPGID YPHTGMEFLQ HVLRPNRRFA PAKLHMDLEV
OV-IA82       IRVGCTVPGE QPFEVIPGID YPHTGMEFLQ HVLRPNRRFA PAKLHMDLEV
B029          IRVGCTVPGE QPFEVIPGID YPHTGMEFLQ HVLRPNRRFA PAKLHMDLEV
                                 o
           201 ▼                 ▼                ▼                250
D1701-B       DYRCVSAVHV KAFLQDACGA RKARTPLYFA GHGSNHPDRR PKNPVPRPQH
D1701-McG     DYRCVSAVHV KAFLQDACSA RKARTPLYFA GHGSNHPDRR PKKPSTAPSA
NZ2           DHRCVSAVHV KAFLQDACSA RKARTPLYFA GHGCNHPDRR PKNPVPRPQH
OV-IA82       DHRCVSAVHV KAFLQDACSA RKARTPLYFA GHGCNHPDRR PKNPVPRPQH
B029          DHRCVSAVHV KAFLQDACSA RKARTPLYFA GHGCNHPDRR PKNPVPRPQH
               :                 .                .               .
              251          265                                     300
D1701-B       VSSPMSRKCC MQTAR~~~~~ ~~~~~~~~~~ ~~~~~~~~~~ ~~~~~~~~~~
D1701-McG     CVVADVQEVL HADSALRALT ALTAVVVCAI AIALEREAEA DAVDLILIKF
NZ2           VSSPISRKCS MQTAR~~~~~ ~~~~~~~~~~ ~~~~~~~~~~ ~~~~~~~~~~
OV-IA82       VSSPISRKCS MQTAR~~~~~ ~~~~~~~~~~ ~~~~~~~~~~ ~~~~~~~~~~
B029          VSSPISRKCS MQTAR~~~~~ ~~~~~~~~~~ ~~~~~~~~~~ ~~~~~~~~~~

              301
D1701-B       ~~~~
D1701-McG     SMIC
NZ2           ~~~~
OV-IA82       ~~~~
B029          ~~~~
```

Supplementary Figure S3B. Comparison of the section of the GIF gene (ORF117) sequence differing between D1701-B and D1701-McG

```
           R  P   K  N  P  V
D1701-B    CGGCC.AAAAAACCCAGTA
D1701-V    CGGCC.AAAAAACCCAGTA
D1701-McG  CGGCCAAAAAAACCCAGTA
           R  P  K  K  P  S
```

Legend S3:

(A) The GIF protein sequences of the D1701 variants were aligned with the indicated ORFV strains (see also Table 2) using program CLUSTAL Omega/MSF. The ▼ marks those aa differing between D1701 and other ORFV. The WSXWS motif is underlined. Completely diverging aa of D1701-McG are shaded grey.

(B) The additional A in D1701-McG results in a frame-shift of the aa sequence of the D1701 sequence published by McGuire et al. [63], and results in complete divergence to the GIF aa of other ORFV strains (see also Fig. S3A).

Supplementary Figure S4. Nucleotide sequence comparison of the core motifs of different poxviral early promoters.

```
pV    AAAATGTAAATACTA
eP1   AAAATTGAAAAATTA
eP2   AAAATTGAAATTCTA
PrS   AAAATTGAAATTTTA
7.5K  AAAgTaGAAAataTA
      *** *  ***   **
E1.1  AAAANTGAAAANNNA
```

Legend S4:

The critical core sequences of the ORFV early promoters used in this study were compared by CLUSTAL Omega with the VACV early promoter PrS [53], the optimized well-known early VACV promoter 7,5K; the bases not important for the promoter strength are written in small letters [54]. Stars indicate identical nt. At the bottom the recently published critical promoter core of VACV immediate early gene class E1.1 [46] is given.
